# Support Exchanges Among Very Old Parents and Their Children: Findings From the Boston Aging Together Study

**DOI:** 10.1093/geroni/igab046.2912

**Published:** 2021-12-17

**Authors:** Kyungmin Kim, Kathrin Boerner, Yijung Kim, Daniela Jopp

**Affiliations:** 1 Seoul National University, Seoul, Seoul-t'ukpyolsi, Republic of Korea; 2 University of Massachusetts Boston, Boston, Massachusetts, United States; 3 The University of Texas at Austin, Austin, Texas, United States; 4 University of Lausanne, Lausanne, Vaud, Switzerland

## Abstract

Very old parents and their “old” children are a growing group in industrialized countries worldwide. Care needs of very old parents can be substantial, while children may also face their own age-related issues. However, little is known about support exchanges within very-old parent-child dyads. This study aimed to identify patterns of support exchanges occurring in these dyads, as well as to ascertain individual and relationship factors associated with these patterns. Participants were 114 very old parents (age ≥ 90) and their children (age ≥ 65) from the Boston Aging Together Study. Data were collected using comprehensive, semistructured in-person interviews with both dyad members, including standardized assessments of support exchanges, relationship quality, health, and perceptions of family norms. Actor-Partner Interdependence Models (APIM) were used to predict upward and downward support reported by children and parents. Both dyad members not only reported substantial upward support (given to parents by children) in all domains but also notable amounts of downward support (given to children by parents) in the domains of emotional support, listening, and socializing. Findings showed significant associations of parent functional impairment, parent and child relationship quality, and child perceptions of family obligation with upward support, and of relationship quality with downward support. Continued support exchanges among very old parents and their children indicated that intergenerational theories still hold up in very late life relationships. Healthcare professionals should be aware that attention to relationship quality and family norms might be vital to ensure that support needs are met.

